# Integrated Analysis of Expression Profile Based on Differentially Expressed Genes in Middle Cerebral Artery Occlusion Animal Models

**DOI:** 10.3390/ijms17050776

**Published:** 2016-05-20

**Authors:** Huaqiang Zhou, Zeting Qiu, Shaowei Gao, Qinchang Chen, Si Li, Wulin Tan, Xiaochen Liu, Zhongxing Wang

**Affiliations:** 1 Department of Anesthesiology, the First Affiliated Hospital of Sun Yat-sen University, Guangzhou 510080, China; liujiaosuan@gmail.com (H.Z.); gscfwid@gmail.com (S.G.); tanwulin1986@163.com (W.T.); 2 Sun Yat-sen University School of Medicine, Sun Yat-sen University, Guangzhou 510080, China; doctorqiuzt@gmail.com (Z.Q.); doctorchenqc@gmail.com (Q.C.); grace980050@gmail.com (S.L.); lxcsysu@gmail.com (X.L.)

**Keywords:** gene expression profile, stroke, middle cerebral artery occlusion, cytokines

## Abstract

Stroke is one of the most common causes of death, only second to heart disease. Molecular investigations about stroke are in acute shortage nowadays. This study is intended to explore a gene expression profile after brain ischemia reperfusion. Meta-analysis, differential expression analysis, and integrated analysis were employed on an eight microarray series. We explored the functions and pathways of target genes in gene ontology (GO) enrichment analysis and constructed a protein-protein interaction network. Meta-analysis identified 360 differentially expressed genes (DEGs) for *Mus musculus* and 255 for *Rattus norvegicus*. Differential expression analysis identified 44 DEGs for *Mus musculus* and 21 for *Rattus norvegicus*. Timp1 and Lcn2 were overexpressed in both species. The cytokine-cytokine receptor interaction and chemokine signaling pathway were highly enriched for the Kyoto Encyclopedia of Genes and Genomes (KEGG) pathway. We have exhibited a global view of the potential molecular differences between middle cerebral artery occlusion (MCAO) animal model and sham for *Mus musculus* or *Rattus norvegicus*, including the biological process and enriched pathways in DEGs. This research helps contribute to a clearer understanding of the inflammation process and accurate identification of ischemic infarction stages, which might be transformed into a therapeutic approach.

## 1. Introduction

Stroke is the second most common cause of death after heart disease. Reduced brain blood flow leads to the loss of the oxygen, glucose, and ATP in cerebral tissue and the depolarization of neurons [1]. Stroke also elevates extracellular potassium concentration and increases oxidative stress. Eccentrically, reperfusion produces the excessive reactive oxygen species (ROS) and leads to additional cerebral injuries, after inflammatory changes and neurotic and apoptotic cell death pathways [2]. Inflammation is the key factor in ischemic stroke, yet the current molecular research concerning the pathways is scarce [3,4].

High throughput technologies can detect the unusual genomic changes among the mass gene expression profiling with microarray. However, previous studies have failed to provide ample samples, and results of the studies are often conflicting and contradictory, which has left their credibility in question. In recent studies, meta-analyses have been employed to detect the differentially expressed genes (DEGs) statistically with an assessment of heterogeneity [5]. In order to overcome the limitations above and generate convincing results, a careful meta-analysis must combine gene expression datasets from multiple sources [6,7].

Recently it was shown that meta-analyses of gene expression data from plenty of sources increased statistical power by pooling effect size with assessment of heterogeneity, and would lead to more powerful, reproducible, and accurate results. Several meta-analysis of microarray data have been reported for a number of central neural system (CNS) conditions. For brain ischemia, single experiments are available, but no coherent meta-analysis has been reported so far. We described an integrated analysis of available data from rodent middle cerebral artery occlusion (MCAO) models and identified molecular signatures and pathways involved in the pathophysiology of stroke in order to overcome the restriction of individual studies and solve inconsistencies among various studies.

## 2. Results

### 2.1. Microarray Data Search and Data Preprocessing

#### 2.1.1. Microarray Data Search

**Figure 1:**
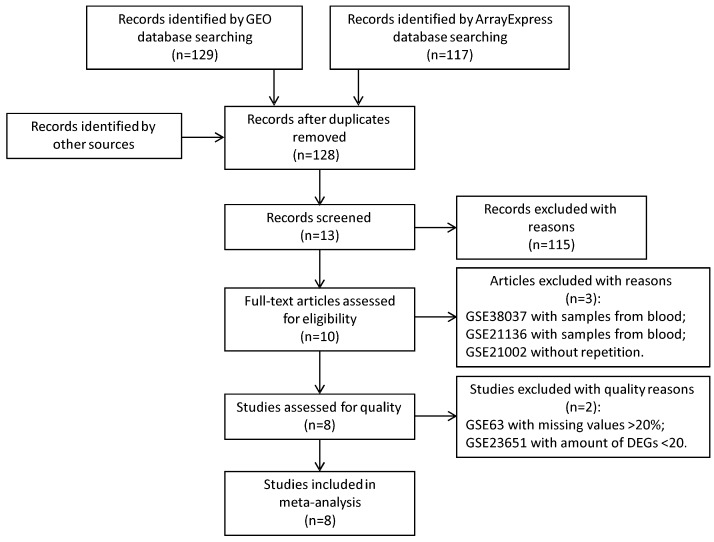
Search in the public microarray data repositories identified 128 microarray datasets. We finally selected eight studies for studies according to the included and excluded criteria.

After a search in the GEO and the ArrayExpress microarray data repositories, we obtained 128 records without duplications and learned that the goals of relevant studies vary according to different learning paradigms, test conditions, subjects, and tissue types. After filtration according to the inclusion and exclusion criteria, eight datasets were finally identified with four *Mus musculus* studies and four *Rattus norvegicus* studies, including a total 36 (*Mus musculus*: 19; *Rattus norvegicus*: 17) sham models and 65 (*Mus musculus*: 39; *Rattus norvegicus*: 26) MCAO models (Figure 1). The table below presents the basic information about the dataset included. Considering there are two species (*Mus musculus* and *Rattus norvegicus*), meta-analysis was performed separately. Characteristics of each study in meta-analysis are presented in Table 1.

#### 2.1.2. Data Preprocessing

Only sham and MCAO arrays were used for further analyses. We carried out probe-to-gene mapping to convert probe-set expression levels into gene expression levels according to the specific chip dataset. After data integration among different chips, there were 13,590 common genes in *Mus musculus* and 11,880 common genes in *Rattus norvegicus*.

### 2.2. Meta-Analysis

We applied five methods to select the genes at the significance level of 0.05. The DEGs identified by at least four methods were selected for further studies. 360 genes (A1: *Mus musculus* DEGs set) were regarded as significantly differentially expressed for four methods while 255 genes (A2: *Rattus norvegicus* DEGs set) in *Rattus norvegicus* fell under the threshold of *p*-value <0.05 for four methods. The following function annotation and pathway enrichment were operated on *Mus musculus* and *Rattus norvegicus* separately and the two species bore some remarkable resemblances.

#### 2.2.1. Mus Function Annotation and Pathway Enrichment Analysis

The response to the molecule of bacterial origin (GO:0002237, *p* = 2.10 × 10^−3^) and regulation of interleukin-12 production (GO:0032655, *p* = 2.46 × 10^−3^) were significantly enriched for biological processes. Calcium ion binding (GO:0005509, *p* = 1.03 × 10^−2^) and growth factor binding (GO:0019838, *p* = 1.21 × 10^−2^) were for molecular function, while cytosolic part (GO:0044445, *p* = 5.75 × 10^−3^) and membrane-enclosed lumen (GO:0031974, *p* = 2.27 × 10^−2^) were for cellular component (Table 2). The most significant pathway was the MAPK signaling pathway (*p* = 8.14 × 10^−5^) and cytokine-cytokine receptor interaction (*p* = 2.65 × 10^−2^) was highly enriched (Table 3).

#### 2.2.2. Rat Function Annotation and Pathway Enrichment Analysis

Response to wounding (GO:0009611, *p* = 1.91 × 10^−7^) and regulation of cell morphogenesis (GO:0022604, *p* = 5.44 × 10^−6^) were significantly enriched for biological processes. Calcium ion binding (GO:0005509, *p* = 5.33 × 10^−5^) and ion channel activity (GO:0005216, *p* = 6.73 × 10^−5^) were for molecular functions, while synapse part (GO:0044456, *p* = 1.44 × 10^−6^) and plasma membrane (GO:0005886, *p* = 1.52 × 10^−6^) (Table 4) were for cellular components. The most significant pathway was in Long-term depression (*p* = 5.93 × 10^−4^). MAPK signaling pathway (*p* = 8.48 × 10^−4^) was also highly enriched.

### 2.3. Differential Expression Analysis in Each Study

We performed differentially expressed analysis between MCAO and sham samples in individual series. Genes were regarded as significantly differentially expressed under the threshold of *p*-value <0.01 and |log2 fold change (FC)| ≥ 1.5. The results were summarized in Table 5. Further study aimed at genes recognized as DEGs by at least three studies. Forty-four DEGs (B1: *Mus musculus* DEGs set) were identified in *Mus musculus*, 21 DEGs (B2: *Rattus norvegicus* DEGs set) in *Rattus norvegicus*. We inputted B1 into the Database for Annotation, Visualization, and Integrated Discovery (DAVID). Chemotaxis (GO:0006935, *p* = 6.10 × 10^−7^) and taxis (GO:0042330, *p* = 6.10 × 10^−7^) were significantly enriched for biological processes. For KEGG pathway detection, the most significant pathway was found in the cytokine-cytokine receptor interaction (*p* = 1.30 × 10^−4^). Furthermore, the Chemokine signaling pathway (*p* = 3.20 × 10^−3^) was highly enriched. However, B2 could not be well described by DAVID for the limitation of the number of B2.

### 2.4. Integrated Analysis

**Figure 2:**
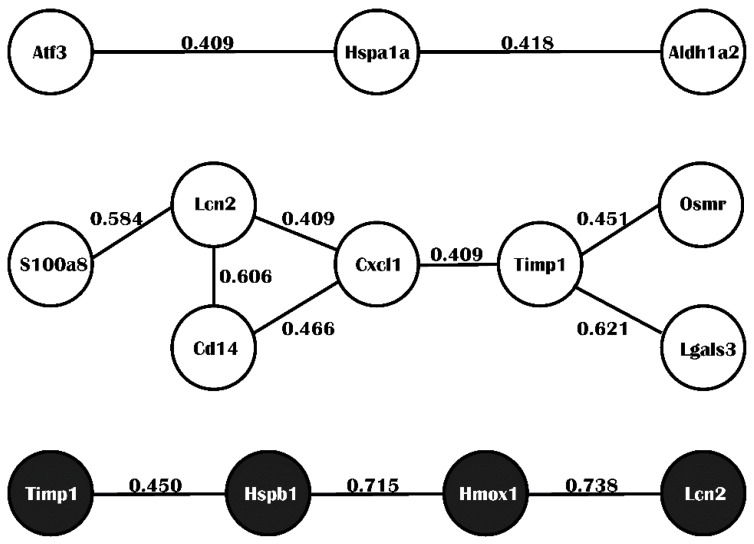
Protein–protein interaction (PPI) network. The confidence scores are marked in the line of interaction. (White: *Mus musculus*; Black: *Rattus norvegicus*).

In integrated analysis, there were 12 common DEGs identified by both individual differential expression analysis and meta-analysis for MCAO *Mus musculus* species, and eight for MCAO *Rattus norvegicus* species. A protein-protein interaction (PPI) network was constructed separately centering on potential hub proteins for *Mus musculus* or *Rattus norvegicus* (Figure 2). Confidence score represented probability of predicted association for both adjacent proteins against a common reference set of functional grouping of proteins maintained in the Kyoto Encyclopedia of Genes and Genomes (KEGG) [16]. We found the common DEGs across species and identified that Timp1 and Lcn2 were both overexpressed in the MCAO *Mus musculus* or *Rattus norvegicus*.

## 3. Discussion

Many genes are expressed differently after cerebral ischemia. A molecular and cellular analysis during stroke is of great importance for clinical management. In the paper, we have performed an integrated analysis using eight publicly available GEO datasets to identify the mechanisms in a stroke. We identified significantly up- or down-regulated genes. We also explored their biological function based on GO enrichment analysis, KEGG pathway analysis, and PPI networks.

Five approaches were applied during meta-analysis. Three hundred and sixty genes were identified as DEGs in *Mus musculus*, and 255 genes in *Rattus norvegicus.* The function annotation and pathway enrichment were operated on *Mus musculus* and *Rattus* *norvegicus* separately and the two species bore some remarkable resemblances. Calcium ion binding was significantly enriched for molecular functions, while MARK signaling pathway was for KEGG pathway analysis. The activation of the p38 MAPK pathway affects the production of pro-inflammatory cytokines such as TNFα, IL-1β, and IL-6 [17]. Roy observed p38 MAPK activation in the glia scar area in a MCAO mouse and suggested that the p38 MAPK signaling pathway might have an impact on the ischemic stroke [18]. Moreover, Chang found that the MAPK signal pathway modulated MMP activity after stroke [19].

We performed differentially expressed analysis between MCAO and sham samples in each study, and obtained the intersection sets of DEG results. Chemotaxis was significantly enriched for biological processes. The cytokine-cytokine receptor interaction and chemokine signaling pathway were highly enriched. Inflammation is important in an ischemic stroke. During a stroke, activation of astrocytes and microglia rapidly strengthen, promote the production of cytokines (including IL-1β, IL-6, and TNF-α and other proinflammatory cytokines) through different pathways such as MAPK signal pathway, induce neutrophils and peripheral lymphocyte infiltration in the focal, then further aggravate the central nervous system injury [20,21,22]. In reports, chemokines were relative to stroke and more researchers regarded them as a new therapeutic target or prognostic indicators. Arac described that interleukin 6 and chemokine (C-C motif) ligand 7, contributed to stroke pathology [23]. A six month follow up study in Chinese stroke patients indicated the association between the high levels of CXC chemokine ligand 12 and unfavorable outcomes and mortality [24]. A blockade of CXCR1/2 chemokine receptors and ELR CXC chemokine antagonism protected mice in ischemic stroke [25,26].

**Figure 3:**
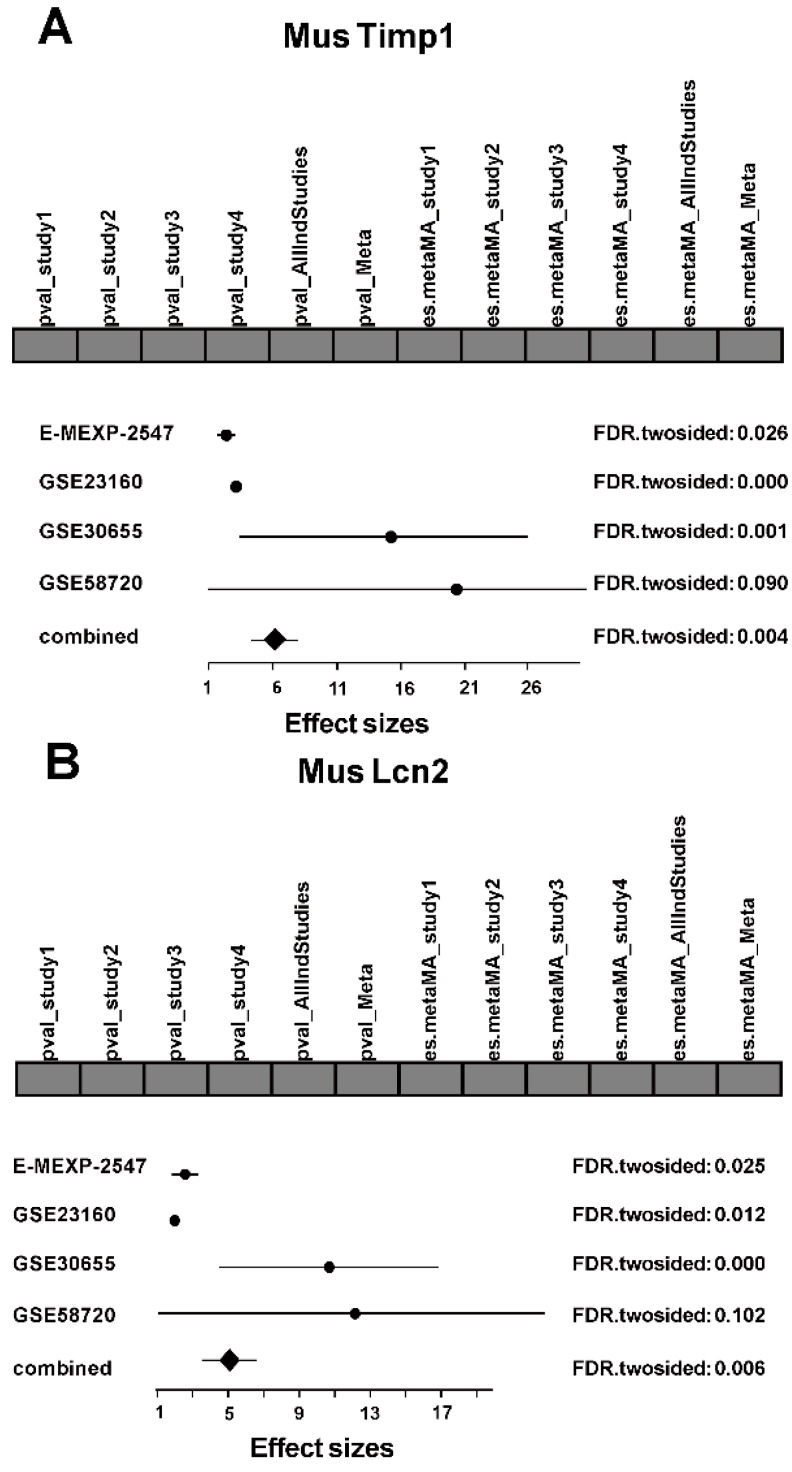

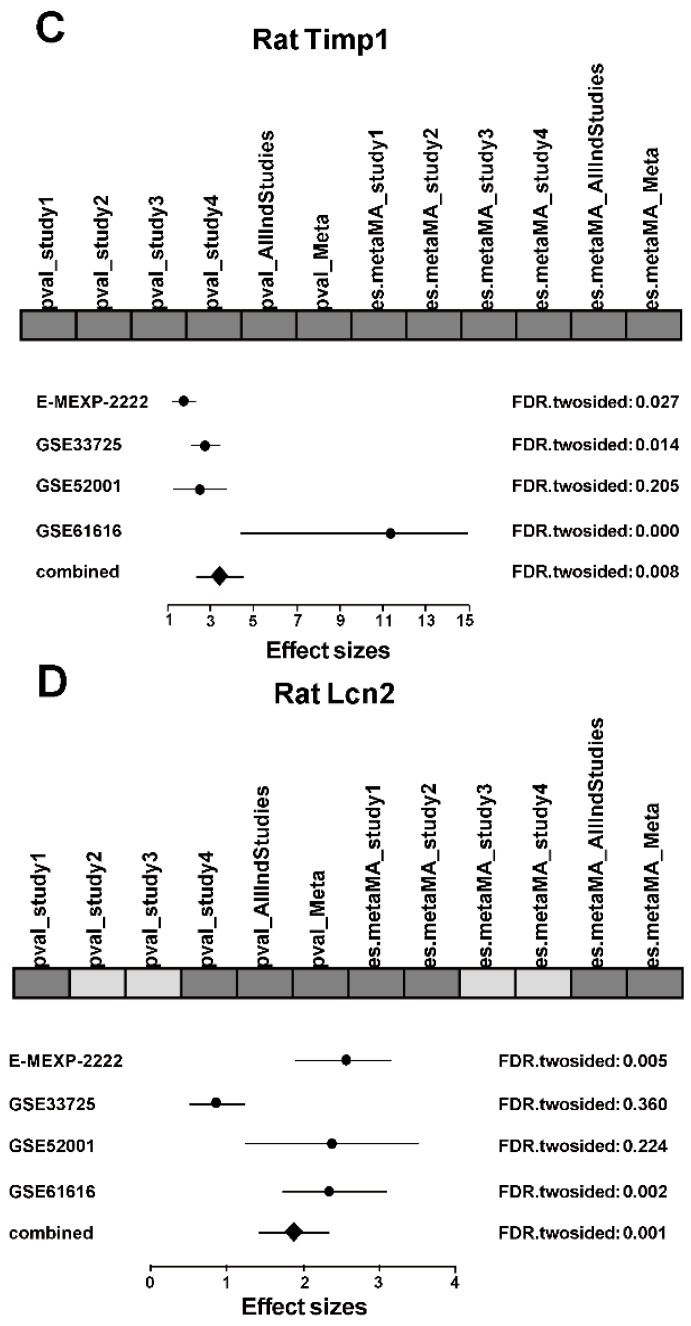
Forest plots of Timp1 and Lcn2. Forest plots of (**A**) Timp1 in *Mus musculus*; (**B**) Lcn2 in *Mus musculus*; (**C**) Timp1 in *Rattus norvegicus*; and (**D**) Lcn2 in *Rattus norvegicus*. For each forest plot, the bar at the top shows the occurrence of combining *p*-values and combining effect size methods in selected genes. The dark gray box means that the result of the particular method in the selected gene is present, while light gray means that it is absent. The graph at the bottom shows the combination of effect size. The point marks the effect size, the horizontal lines denote the variance of effect size. Adjusted FDR (false discovery rate) on the right side of the graph presents the statistical significance of the difference in gene expression.

Timp1 and Lcn2 were regarded as common significant DEGs across species when we performed integrated analysis, either of which was observed to be over-expressed in MCAO mice (Figure 3). Timp1, the TIMP metallopeptidase inhibitor 1, is a glycoprotein against most of the known MMPs, and may have an anti-apoptotic function [27]. Wang utilized a subtractive cDNA library strategy in focal strokes and identified Timp1 with increased gene expression [28]. The relationship between Timp1 and MMPs is intriguing. Specifically, the MMP9/ Timp1 was elevated on the seventh day of stroke, which might provide a biomarker of prognosis [29,30]. Considering what we mentioned before, MAPK signal pathway modulates MMP activity after a stroke. In recent years, more studies are engaged in the relationships between Timp1 and MAPK pathways [19]. Overexpression of endogenous Timp1 activates ERK1/2 and JNK1/2 of the MAPK signaling pathway [31]. p38 MAPK inhibitors block all natural haemozoin effects on Timp1 and proinflammatory molecules [32]. Lcn2, neutrophil gelatinase-associated lipocalin (NGAL) sequestrates iron and hinders bacterial growth [33,34]. Recently, rapidly elevated Lcn2 levels have been reported in the plasma of ischemic stroke patients, but its role in strokes is unknown [35,36]. Jin indicated that Lcn2 might contribute to the death of neuronal cells in the ischemic brain by promoting neurotoxic glial activation, neuroinflammation, and blood-brain barrier disruption [36,37]. It suggests that Lcn2 is a potential early stroke biomarker and a novel therapeutic target to reduce injury. In order to illustrate its molecular function in ischemia and reperfusion, more research needs to be conducted.

Except Timp1, CXCL1 is the hub of the PPI network. CXCL1 belongs to the CXC chemokine family and was previously called GRO1 oncogene, GROα [38]. CXCL1 is expressed by macrophages, neutrophils, and epithelial cells in neutrophil chemoattractant activity, inflammation, wound healing, and angiogenesis [39,40]. It elicits its effects by signaling CXCR2 [41]. In 2005, Losy observed that there was higher CXCL1 in cerebrospinal fluid level than the control group in stroke patients, and suggested the potential neutrophil chemoattractant function in the pathophysiology of stroke [42]. No new evidence had surfaced to illustrate it, until our study confirmed the finding of Losy at the gene expression level.

Undeniably, our current study has many limitations. First, different studies might be heterogeneous due to the variation of microarray platforms, labs, technicians, and so on. Heterogeneity and confounding factors may be interfering with the analysis, even under the circumstance that we applied strict criteria to the studies included in order to minimize the potential for errors; Second, there are differences in gene expression between species such as *Mus musculus* and *Rattus norvegicus*. Attempting to minimize the influence, we analyzed them separately before we integrated the analyses to detect common important genes in the species; Third, a number of samples did not provide details of different periods. As a result, we were unable to do a meta-analysis of expression profiles along with the time of developing; Fourth, meta-analysis, differential expression analysis, and integrated analysis were performed to reduce heterogeneity owing to various microarray platforms instead of putting all the expression matrix together and then performing differential expression analysis [43].

## 4. Materials and Methods

### 4.1. Microarray Data Search

We searched the Gene Expression Omnibus (GEO, available at: http://www.ncbi.nlm.nih.gov/geo/) and the ArrayExpress (available at:http://www.ebi.ac.uk/arrayexpress/) microarray data repositories for microarray gene expression datasets by using the keywords “brain ischemia reperfusion”, “stroke”, and “MCAO”. We also used the literature database PubMed and Embase to search for relevant studies. The available raw data were downloaded from the GEO and ArrayExpress public databases.

### 4.2. Included and Excluded Criteria

The eligible criteria were as follows: (1) the examined samples were tissue section of ipsilateral cerebral cortex; (2) the data were on gene expression level; (3) the species were *Rattus norvegicus* or mice; (4) the microarray platform was limited to single channel; (5) the occlusion in the trial group must be in the middle cerebral artery and sham groups must undergo the same surgery but without real occlusion. The exclusion criteria were as follows: (1) samples were from other tissues or *vitro* assays; (2) datasets were not the original gene expression level data files; (3) the data were with redundant subdatasets; (4) samples were without at least three repetitions in an experiment; (5) the samples had permanent ischemia reperfusion; (6) the data had only miRNA profiles; (7) the series had low quality, such as containing more than 20% missing values on arrays.

### 4.3. Data Preprocessing

The data were analyzed by R software (available at: http://www.R-project.org/) and packages in Bioconductor (available at: http://www.bioconductor.org/). We used the visual inspection for image quality, relative log expression (RLE), and normalized unscaled standard errors (NUSE) for data quality and affyPLM package for RNA degradation. We only included the samples with high quality and the independent decision by the original authors [44]. The expression data were processed using the limma package or affy package in R software, including background correction, quantile normalization, and final probe summarization [45,46]. Missing values were filled by using the k-nearest neighbors (KNN) method based on the average of non-missing neighboring values of its neighbor. For each probe-set, intensity values were log2-transformed and normalized to 0 mean and unit variance [47]. Lastly, we carried out probe-to-gene mapping to convert probe-set expression levels into gene expression levels according to the specific chip dataset [48]. If multiple probes mapped to a gene, the mean of the probe effect size was selected.

### 4.4. Meta-Analysis

For either *Rattus norvegicus* or *Mus musculus*, we merged the common gene-set among the studies before meta-analysis. The original data sets have been preprocessed and subsampled to reduce the computational complexity of the meta-analysis. Considering there were cross species (*Mus musculus* and *Rattus norvegicus*), we performed meta-analysis separately. Using MAMA package, five approaches had been utilized to select the genes at the significance level of 0.05, including (1) combine *p*-values in sense of sum of logs by Fisher′s *S*-statistic; (2) combine effect sizes by classical and moderated *t*-test and random-effect model [49]; (3) non-parametric statistic of RankProduct that detects up-regulated and down-regulated genes under one condition against another condition [50]; (4) *Z*-statistic utilizing posterior mean differential expression weighted by variances; (5) combine effect sizes by Hedge’s and Olkin’s g method. The DEGs were recorded by at least four of five approaches, which were further researched in function annotation and pathway enrichment analysis.

### 4.5. Differential Expression Analysis in Each Study

Using the limma package, false discovery rate correction was performed and we selected DEGs with the criteria of both *p*-value < 0.05 and |log2fold change (FC)| ≥ 1.5. Considering there are cross species (*Mus musculus* and *Rattus norvegicus*), we performed differential expression analysis separately and identified the common DEGs in at least three of four series for further exploration. Venn diagrams were constructed to display the intersection of DEGs results.

### 4.6. Integrated Analysis

**Figure 4:**
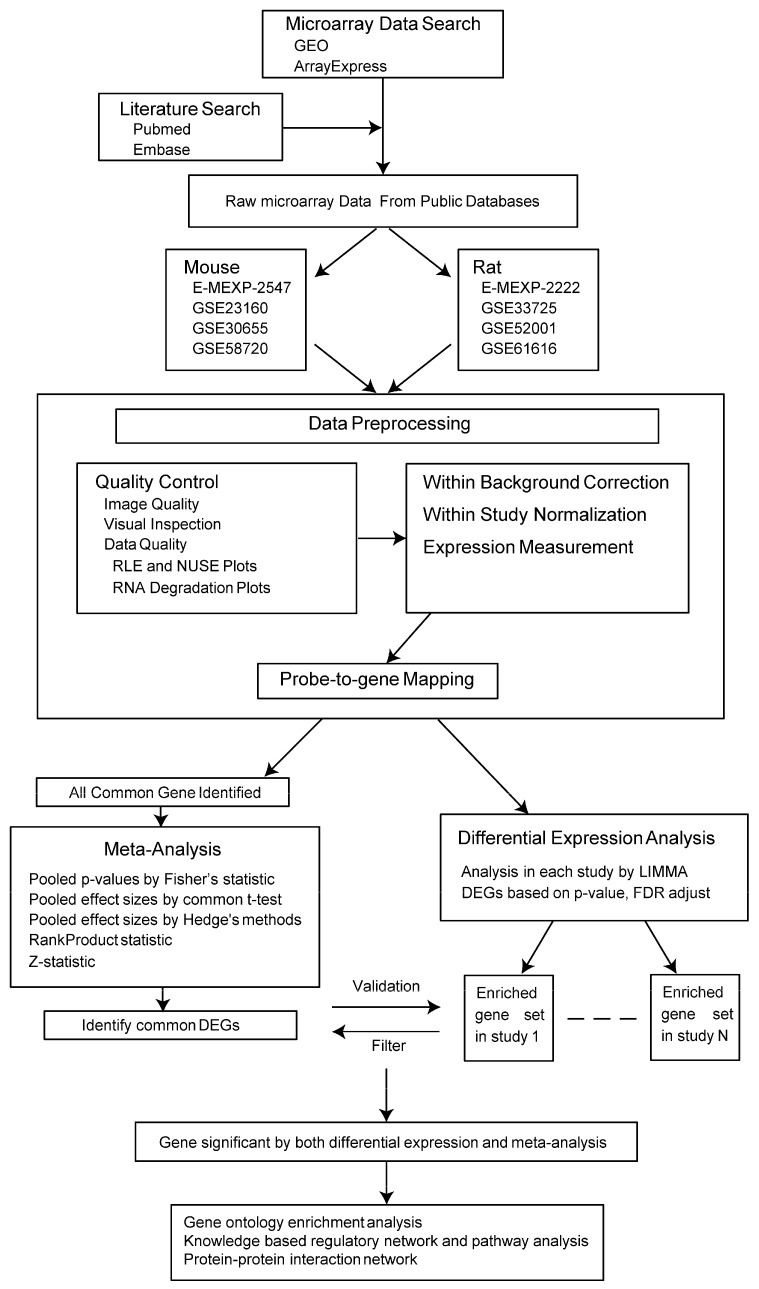
A summary of analysis workflow. RLE, relative log expression; NUSE, normalized unscaled standard errors; DEGs, differentially expressed genes. Solid line with arrow refers to directed workflow. Dash line refers to enriched gene sets of several studies.

The individual datasets are useful for validation to show that genes identified from meta-analysis show up in the individual datasets and are concordant in expression change. To elevate the positive predictive power, we entered DEGs from meta-analysis into each microarray. Thus, more specific DEGs were filtered when they were significantly expressed in at least three individual datasets (there were four datasets in total). Next, the potential hub proteins were sought through the protein-protein interaction network based on the DEGs above. The overall analysis workflow is depicted in Figure 4.

### 4.7. Functional Annotation

Functional and pathway analyses were performed mainly using the Database for Annotation, Visualization, and Integrated Discovery (DAVID, available at: https://david.ncifcrf.gov/). To understand the significance of genes, we performed the Gene Ontology (GO) classification, making use of the following categories: BP_Fat (biological process), CC_Fat (cellular component), and MF_Fat (molecular function) [51]. We also performed the Kyoto Encyclopedia of Genes and Genomes (KEGG) pathway enrichment analysis to detect the potential pathway of target genes and had the hypergeometric test with *p* value <0.05 [52]. STRING version 10.0 (available at: http://string-db.org/) comprises of >1100 completely sequenced organisms [53]. To identify the interactive associations among genes, the DEGs were input into STRING. The Cytoscape v2.8.0 software was used to visualize these associations and the mined modules [16].

Data were integrated across studies and across array platforms after data preprocessing and analyzed in several ways: meta-analysis and individual differential expression analysis and integrated analysis.

## 5. Conclusions

In conclusion, through multiple analyses based on gene expression, we have exhibited a global view of the potential molecular differences between MCAO model and sham for *Mus musculus* or *Rattus norvegicus*, including DEGs and their enriched pathways. This research helps contribute to the further understanding of the pathophysiology of stroke involving inflammation process and accurate identification of different stages of ischemic infarction, which might be transformed into a therapeutic approach.

## Figures and Tables

**Table 1:** Characteristics of included studies analyzed in the meta-analysis.

Series Dataset	Ischemic Period (min)	Reperfusion Period (h)	No. of Samples		Platform
			MCAO	Sham	
*Mus musculus*					
E-MEXP-2547 [8]	30	24, 240	5	5	Affymetrix GeneChip Mouse Gene 1.0 ST Array
GSE23160 [9]	120	2, 8, 24	24	8	Illumina MouseRef-8 v2.0 expression beadchip
GSE30655 [10]	60	24	7	3	Affymetrix Mouse Genome 430 2.0 Array
GSE58720 [11]	90	24	3	3	Agilent-028005 SurePrint G3 Mouse GE 8 × 60 K Microarray
*Rattus norvegicus*					
E-MEXP-2222 [12]	90	6, 24	12	3	Affymetrix Rat Genome 230 2.0 Array
GSE33725 [13]	NA	2, 6	6	6	Agilent-014879 Whole Rat Genome Microarray 4 × 44 K G4131F
GSE52001 [14]	120	144	3	3	Agilent-028282 Whole Rat Genome Microarray 4 × 44 K v3
GSE61616 [15]	120	168	5	5	Affymetrix Rat Genome 230 2.0 Array

GEO, gene expression omnibus; GSE, gene expression series; MCAO, middle cerebral artery occlusion; No, number; NA, not available.

**Table 2:** Gene ontology (GO) functional annotation for differentially expression genes in *Mus musculus* (Top five).

GO ID	GO Term	%	*p*-Value
**Biological Process**			
GO:0002237	response to molecule of bacterial origin	1.67	2.10 × 10^−3^
GO:0032655	regulation of interleukin-12 production	1.11	2.46 × 10^−3^
GO:0043086	negative regulation of catalytic activity	2.22	2.78 × 10^−3^
GO:0010033	response to organic substance	5.56	2.92 × 10^−3^
GO:0044092	negative regulation of molecular	2.50	3.28 × 10^−3^
**Cellular Component**			
GO:0044445	cytosolic part	1.67	5.75 × 10^−3^
GO:0031974	membrane-enclosed lumen	8.89	2.27 × 10^−2^
GO:0005829	cytosol	5.00	2.33 × 10^−2^
GO:0005886	plasma membrane	18.61	2.59 × 10^−2^
GO:0005938	cell cortex	1.94	2.78 × 10^−2^
**Molecular Function**			
GO:0005509	calcium ion binding	7.50	1.03 × 10^−2^
GO:0019838	growth factor binding	1.67	1.21 × 10^−2^
GO:0043167	ion binding	25.28	2.49 × 10^−2^
GO:0046872	metal ion binding	24.72	2.75 × 10^−2^
GO:0003677	DNA binding	12.78	2.92 × 10^−2^

**Table 3:** List of the top five pathways based on Kyoto Encyclopedia of Genes and Genomes (KEGG) analysis.

ID	Term	%	*p*-Value
***Mus musculus***			
mmu04010	MAPK signaling pathway	4.72	8.14 × 10^−5^
mmu04060	Cytokine-cytokine receptor interaction	3.06	2.65 × 10^−2^
mmu04062	Chemokine signaling pathway	2.50	3.18 × 10^−2^
mmu04722	Neurotrophin signaling pathway	1.94	4.90 × 10^−2^
mmu05410	Hypertrophic cardiomyopathy (HCM)	1.39	9.16 × 10^−2^
***Rattus norvegicus***			
rno04730	Long-term depression	2.77	1.44 × 10^−6^
rno04010	MAPK signaling pathway	5.14	1.52 × 10^−6^
rno04070	Phosphatidylinositol signaling system	2.37	1.82 × 10^−6^
rno04540	Gap junction	2.37	2.29 × 10^−6^
rno04670	Leukocyte transendothelial migration	2.77	3.29 × 10^−6^

**Table 4:** GO functional annotation for differentially expressed genes in *Rattus norvegicus*. (Top five).

GO ID	GO Term	%	*p*-Value
**Biological Process**			
GO:0009611	response to wounding	9.49	1.91 × 10^−7^
GO:0022604	regulation of cell morphogenesis	4.74	5.44 × 10^−6^
GO:0006954	inflammatory response	5.53	3.57 × 10^−5^
GO:0006813	potassium ion transport	4.35	7.65 × 10^−5^
GO:0006811	ion transport	9.88	1.16 × 10^−4^
**Cellular Component**			
GO:0044456	synapse part	6.72	1.44 × 10^−6^
GO:0005886	plasma membrane	24.90	1.52 × 10^−6^
GO:0043005	neuron projection	8.70	1.82 × 10^−6^
GO:0005856	cytoskeleton	13.44	2.29 × 10^−6^
GO:0045202	synapse	7.91	3.29 × 10^−6^
**Molecular Function**			
GO:0005509	calcium ion binding	9.49	5.33 × 10^−5^
GO:0005216	ion channel activity	6.32	6.73 × 10^−5^
GO:0043167	ion binding	25.30	8.37 × 10^−5^
GO:0022838	substrate specific channel activity	6.32	9.35 × 10^−5^
GO:0022836	gated channel activity	5.53	9.48 × 10^−5^

**Table 5:** Summary of differential expression analysis in each study.

Study	Count	Up-Regulated	Down-Regulated
***Mus musculus***			
E-MEXP-2547	179	179	0
GSE23160	22	22	0
GSE30655	341	128	213
GSE58720	1162	841	321
***Rattus norvegicus***			
E-MEXP-2222	83	81	2
GSE33725	38	38	0
GSE52001	94	66	28
GSE61616	827	695	132

Count = the number of differentially expressed genes.

## Abbreviations

GO	Gene ontology
DEGs	Differentially expressed genes
ROS	Reactive oxygen species
CNS	Central neural system
RLE	Relative log expression
NUSE	Normalized unscaled standard errors
KNN	k-nearest neighbors
DAVID	Database for Annotation, Visualization and Integrated Discovery
KEGG	Kyoto Encyclopedia of Genes and Genomes
PPI	Protein-protein interaction
NGAL	Neutrophil gelatinase-associated lipocalin
MCAO	Middle cerebral artery occlusion
